# Protocol for systematic reviews of determinants/correlates of obesity-related dietary and physical activity behaviors in young children (preschool 0 to 6 years): evidence mapping and syntheses

**DOI:** 10.1186/2046-4053-2-28

**Published:** 2013-05-10

**Authors:** Rajalakshmi Lakshman, Veena Mazarello Paes, Kathryn Hesketh, Claire O’Malley, Helen Moore, Ken Ong, Simon Griffin, Esther van Sluijs, Carolyn Summerbell

**Affiliations:** 1 MRC Epidemiology Unit, Addenbrookes Hospital, Box 285, Cambridge CB2 0QQ, UK; 2 UKCRC Centre for Diet and Activity Research (CEDAR), Institute of Public Health, Box 296, Cambridge, CB2 0SR, UK; 3 Cambridge Institute of Public Health, Cambridge, CB2 0SR, UK; 4 Obesity Related Behaviours Research Group, Durham University, Stockton on Tees, TS17 6BH, UK

## Abstract

**Background:**

The aim of these reviews is to inform the design and content of interventions to reduce obesity in young children. The behaviors that are associated with obesity/overweight have been studied extensively; however, the factors associated with these behaviors in young children (0 to 6 years) have not been systematically reviewed. Over the past few years the focus of obesity prevention has shifted to preschool children because of the high prevalence of obesity at school entry and recognition that habits formed in early life could track into adulthood. In order to develop effective interventions and change behavior, it is important to understand the factors that are associated with those behaviors. For example, we need to understand whether it would be more important to target the family, childcare settings or the wider environment and identify the most effective way of changing these energy balance related behaviors.

**Methods/Design:**

Quantitative (intervention and observational) and qualitative literature on determinants/correlates of fruit and vegetable intake, sugar sweetened beverage and other unhealthy diet intake, and physical activity and sedentary behaviors in young children will be systematically identified, mapped and reviewed. A common search strategy (no language or period restrictions) will be used to identify papers from eight electronic databases and this will be supplemented by hand-searching. Next, studies in developed countries that examine the factors associated with these behaviors in children aged 0 to 6 years (at baseline) will be screened and mapped descriptively followed by in-depth data extraction, quality assessment and synthesis. Data from quantitative studies will be summarized using either forest plots or harvest plots and narrative synthesis, and qualitative studies using thematic analysis. Qualitative evidence will be integrated with the quantitative evidence, using a parallel synthesis approach, to provide a deeper understanding of effective strategies to influence these energy balance related behaviors.

**Discussion:**

In addition to updating and mapping current research, these reviews will be the first to comprehensively synthesize and integrate both the quantitative and qualitative evidence pertaining to determinants/correlates/barriers/facilitators of obesity related behaviors in this young age group (0 to 6 years) with the aim of informing future interventions.

**Trial registration:**

International Prospective Register for Systematic Reviews (PROSPERO) Registration number: CRD42012002881

## Background

The prevalence of childhood overweight and obesity has increased 2- to 4-fold between 1980 and 2000 in developed countries [1], although levels have started to plateau [2-4]. Obesity is increasing even in preschool children and, in 2010, 43 million preschool children (35 million in developing countries) were overweight or obese [5]. The rising prevalence of childhood obesity therefore presents a major public health challenge for the 21st century, increasing the burden of chronic non-communicable diseases in both developed and developing countries [6].

The preschool years are a period of rapid growth and habit formation. Hence, it is possible that at least some of the solutions to the obesity epidemic will be found here, yet few interventions have been developed for preschool children. Recent systematic reviews of obesity prevention interventions in preschool children have concluded that the evidence base for interventions in this age group was sparse [7,8]. The 2011 updated Cochrane review on interventions for preventing obesity in children included 55 studies, only eight of which were in children aged 0 to 5 years, yet these studies showed the largest intervention effects [9].

**Figure 1:**
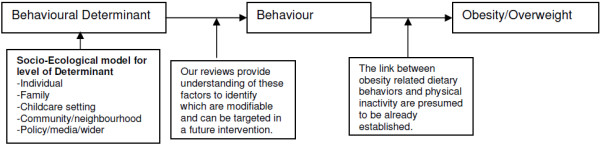
Schematic diagram showing focus of review.

In the simplest sense, obesity is an imbalance between energy intake and energy expenditure over a long period of time. At an individual level, behavior is key in influencing energy intake (mainly diet) and energy expenditure (mainly physical activity). It is therefore important to understand the behaviors that lead to obesity and the factors that are associated with such behaviors. Interventions to improve health-related behaviors targeting the most important determinants/correlates of these behaviors are more likely to be effective [10,11]. The socio-ecological model [12] provides a useful framework for defining the level of determinant or correlate: individual (for example, gender, ethnicity); family (for example, parenting style, single parent, siblings); childcare setting/preschool (for example, school policies); community/neighborhood (for example, food outlets, parks, safety); and policy/media/wider (for example, campaigns, taxation). Use of this framework will allow us to understand whether it would be more important to target the family, childcare settings or the wider environment and what would be the most effective way of doing this (see Figure 1 for schematic diagram outlining the scope of the review).

A number of systematic reviews have been conducted examining the effects of diet and physical activity on the development of obesity and chronic diseases; however, few reviews have focused on the more upstream determinants/correlates of these behaviors in young children. Where such reviews have been conducted, the results have been inconsistent due to different inclusion criteria (age range, study period, study populations) [13-15] and no review to date has attempted to combine observational, intervention and qualitative evidence to provide a comprehensive overview of the literature on determinants/correlates of obesogenic behaviors in children aged 0 to 6 years.

Hence this work aims to comprehensively review the quantitative (observational and intervention) and qualitative literature on determinants/correlates of fruit and vegetable intake, sugar-sweetened beverage and other unhealthy diet intake, and physical activity and sedentary behaviors in preschool children. Secondary questions include: 1) which of these determinants/correlates are modifiable and can therefore be addressed through interventions; 2) should interventions be focused at the individual, family, childcare setting, community or policy level; and 3) what are the gaps in the existing literature and areas for future research?

## Methods/Design

The methods follow established criteria for the rigorous conduct and reporting of systematic reviews [16]. For each of the three topic areas (fruit and vegetable intake, sugar sweetened beverages and unhealthy diet intake, and physical activity and sedentary behavior) the reviews will be carried out in three stages as described in the Evidence for Policy and Practice Information (EPPI) and coordinating centre reviews on barriers and facilitators to fruit and vegetable intake and physical activity in 4- to 10-year-old children [17,18].

Stage 1 involves searching of bibliographic databases, application of broad inclusion and exclusion criteria. and synthesis of a descriptive map of the number, types and quality attributes of existing research studies.

Stage 2 will take a subset of high-quality quantitative studies identified in Stage1 for detailed data extraction and in-depth synthesis.

Stage 3 will involve a thematic analysis of the qualitative literature to be integrated with the findings of the quantitative literature using a parallel synthesis approach as used previously [19]. Using the evidence from the qualitative research to explain the quantitative findings will provide a deeper understanding of effective strategies to influence these energy balance related behaviors.

### Search strategy

As many studies are common to the three review topics, the database searches, processes of developing criteria for including studies and identifying and classifying studies were run in tandem for all the reviews (by HM). A common search strategy was used to identify papers from eight electronic databases – Medline, Embase (via OVID), Cinhal, Psychinfo (via Ebsco), Web of Knowledge (via Thomson Reuters), British Nursing Index (BNI), Applied Social Sciences Index and Abstracts (ASSIA) and Sociological Abstracts (via Proquest).

A number of initial scoping searches were carried out to refine the search strategy to maximize sensitivity and specificity. This included contacting experts in the field and identifying key publications followed by running the searches and ensuring that important publications were captured. The four sets of search terms relate to the population (young children), exposure (terms to capture observational, intervention, qualitative studies and review articles), outcome (diet and physical activity) and to exclude clinical populations (see Additional file 1 for search strategy). Since previous reviews showed that restricting searches to certain time periods resulted in conflicting conclusions [13], no time period or language restrictions were applied. All identified articles were imported into an Endnote Database and de-duplicated. This will be supplemented by hand-searching references of included articles and relevant reviews.

### Inclusion criteria

Observational (non-intervention) longitudinal (prospective and retrospective) and intervention (randomized control trials (RCTs) and non-RCTs) studies that quantify the association between a risk factor/correlate/determinant AND obesity-related dietary behavior or physical activity or sedentary behavior in children aged 0 to 6 years at baseline will be included. Objectively (diet diaries, food records, 24-hour recalls, accelerometers, combined heart-rate monitors and pedometers) and subjectively (self-report, questionnaires) measured outcomes will be included. Qualitative studies that provide greater in-depth understanding of barriers and facilitators of these behaviors will also be included. Studies of obese participants will be included. Only studies that focus on investigating correlates (cross-sectional association) or determinants (prospective association) of the relevant behaviors will be included, and studies that merely present a descriptive table in an otherwise unrelated study will not be included.

### Exclusion criteria

The following are exclusion criteria: non-human studies; cross-sectional studies (only to be included in absence of other high-level evidence); laboratory-based studies (such as the vitamin and preloading diets); studies on health outcomes for these behaviors (for example, studies describing the association between sedentary behavior and obesity or cardiovascular risk factors); quantitative studies that measure these behaviors but do not describe an association with any other variables; studies in clinical populations (for example, malnutrition, disability, allergy, dental caries, asthma, cerebral palsy, cystic fibrosis, autism etc.); studies on breast/bottle feeding and weaning of infants.

### Study selection procedure

Three reviewers (KH, CO, VP) will undertake title and abstract screening in small batches (n = 500) based on a piloted screening protocol. One senior reviewer (CS) will screen the same studies and compare results until there is less than 5% discrepancy, after which screening will be done individually [20]. An overall 10% of the total articles will be randomly selected and double screened by two additional reviewers (RL, EvS). Papers which meet the inclusion criteria will be ordered for full review. Specific study details such as study design, country of study, study population, exposures assessed, outcome assessed and other valuable information will be extracted to an IN/OUT spreadsheet/form. At this stage, cross-sectional studies will not be excluded. Based on this information, an evidence map of the existing literature will be created (stage 1). Any disagreement will be resolved by discussion and re-examination of the article. All studies meeting the inclusion criteria will be sorted for further review and data extraction, according to the research question or the behavior being investigated.

### Evidence mapping

A descriptive map of the evidence will be created to highlight gaps in the evidence base, but also to identify where sufficient data exist to warrant a review. Data will be synthesized on countries the studies came from, publication year, study design (for example, cross-sectional, longitudinal, intervention, qualitative), behavior studied (for example, fruit and vegetable intake, sugar-sweetened beverage or other unhealthy diet intake, physical activity or sedentary behavior) and type and level of determinant studied (for example, individual, family, childcare, community or wider).

### Data extraction

Three reviewers (KH, CO, VP) will systematically review the studies pertaining to the allocated research question or behavior. A data extraction form/spreadsheet has been piloted to ensure consistency of data extraction across reviews and reviewers. Data will be extracted into the spreadsheet by one reviewer and a proportion of the studies will be double-reviewed by a second reviewer (RL, EvS, CS). Discrepancies will be resolved by discussion within the review team. For longitudinal studies (observational or intervention), the latest data available before the children are 6 years old will be included. The data extraction form will be organized to specify the level of determinant - individual, family, childcare, community or wider (See Additional file 2 for details of data extracted).

### Quality assessment

Study quality will be systematically assessed against preset quality criteria using standard quality assessment tools specified for the respective study designs and used by the EPPI centre as follows.

#### Intervention studies

The following criteria will be assessed in intervention studies: randomization, effect of intervention reported for all outcomes, pre-intervention data on all outcomes, post-intervention data on all outcomes, allocation concealment, blinding, objective measurement of outcome, and retention >70%.

#### Non-intervention studies

For non-intervention studies we will be looking at the following criteria: number of participants, representativeness/generalizability, prospective data collection (versus cross-sectional), multivariate analyses (versus univariate), objective (versus subjective) measure of exposure, and objective measure of outcome.

#### Qualitative studies

In qualitative studies, the following set criteria will be investigated: research questions clearly stated, approach appropriate for the research question, qualitative approach clearly justified, study context clearly described, role of the researcher clearly described, sampling method clearly described, sampling strategy appropriate for the research question, method of data collection clearly described, data collection method appropriate, method of analysis clearly described, analysis appropriate for the research question, and conclusions supported by sufficient evidence.

### Data synthesis

Narrative and, where possible, statistical data synthesis will be undertaken.

#### Intervention studies

Where possible, an attempt will be made to meta-analyze the data and present results as a Forest-plot [21], only including randomized trials of high-quality studies with a low risk of bias.

#### Non-intervention studies

Where it is not possible to synthesize the data using Forest plots, Harvest plots [22] will be used. Direction and strength of the association will be summarized using the following symbols: significant negative association, - -; non-significant negative association, -; null association, 0; non-significant positive association, +; significant positive association, + +. Results for categorical and continuous outcome variables will be consistently recoded so that a single or double + always denotes higher risk for the undesirable behavior, and a single or double - always denotes a lower risk for the undesirable behavior. These data will then be displayed using bar charts as follows:

1. Position based on direction and strength of association (++, +, 0, -, --).

2. Height of bar representing size of study.

3. Color of bar representing quality: black, dark grey and light grey with darker bars representing higher quality studies.

4. Symbol on top for study identification.

Conclusions will be drawn based on the consistency of results of studies of the highest available quality level, consistency defined as >75% of results being in the same direction [23].

#### Qualitative studies

Thematic synthesis will be used to summarize qualitative studies. The qualitative findings will be integrated with the quantitative findings using the parallel synthesis approach recommended for mixed-methods research synthesis [17]. Themes identified in the qualitative studies will be used to interpret the findings of the quantitative studies and recommendations will be drawn for future interventions. For example, a review of barriers and facilitators to fruit and vegetable intake in 4- to 10-year-old children concluded that, although the qualitative evidence suggested that branding fruit and vegetables as ‘tasty’ rather than ‘healthy’ and making the messages salient to children and the social context were important, few interventions used these strategies [17].

## Discussion

**Figure 2:**
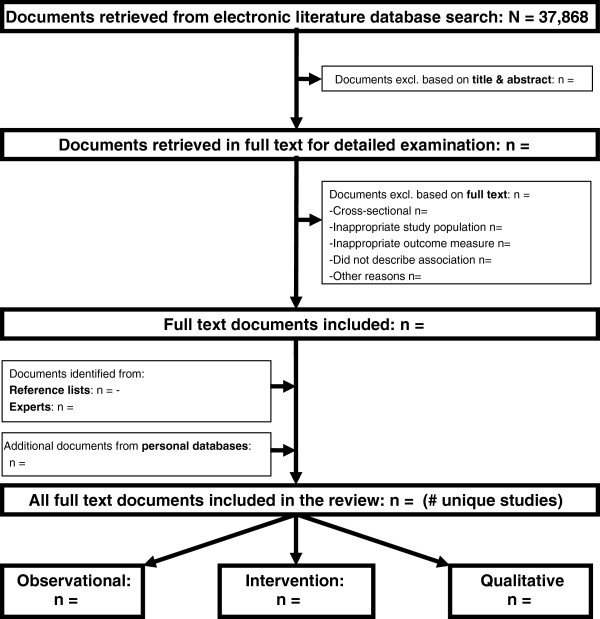
Flowchart presenting an overview of the search results.

As far as we are aware, these reviews will be the first to integrate, in a rigorous and systematic way, the findings of quantitative (both observational and intervention studies) and qualitative research on this topic. It is considered that this type of synthesis provides a more complete and trustworthy picture than relying on syntheses of any one type of research in isolation [17]. After de-duplication, 37,868 papers were downloaded to an Endnote database (Medline, n = 20374; Embase, n = 17331; Cinahl, n = 775; Psychinfo, n = 1868; Sociological Abstracts, n = 135; ASSIA, n = 113; Web of Knowledge, n = 13455; and BNI, n = 291). Figure 2 demonstrates a flow diagram of the study selection process.

### Comparison with previous reviews in young children

Most reviews have examined obesity prevention interventions. A Health Technology Assessment (HTA) systematic review in 2009 [7] and a 2010 systematic review [8] found an absence of effective interventions to prevent obesity in preschool children. The HTA review only included studies that reported body mass index (BMI) as an outcome and identified three studies. The 2010 review included studies reporting BMI, diet or physical activity as outcomes and identified 23 studies. Both reviews concluded that the evidence base for obesity prevention in preschool children was sparse and further research was urgently needed with well designed RCTs in preschool children. More recently, the ToyBox Study Group have published a series of systematic reviews relating to childcare-based (preschools/schools) interventions to prevent obesity in children aged 4 to 6 years [24]. Those reviews included educational strategies [25], psychological approaches [26] and behavioral models [27] underpinning interventions targeting diet and physical activity in the preschool/school setting, and recommended that childhood obesity was not an issue for the education sector alone, but needed to be tackled at a multi-sectoral level [28]. Our reviews on the behavioral determinants/correlates will provide further evidence on the levels (individual, family, preschool, community or wider) at which interventions could be effectively targeted.

Previous systematic reviews that examined the correlates of physical activity in 2008 [15] and sedentary behavior in 2010 [14] in 2- to 5-year-old children found that male sex, having active parents and spending more time outdoors was associated with higher physical activity, but the evidence for sedentary behavior was inconclusive. A more recent systematic review in 2011 [13] of correlates of energy balance-related behaviors in 4- to 6-year-old children concluded that gender, age and socioeconomic status were not associated with physical activity, while an indeterminate result was found for ethnicity. Gender and ethnicity were not associated with sedentary behavior and indeterminate results were found for age and socioeconomic status. Watching television was associated with a higher consumption of snacks and sugar-sweet beverages [13].

There are no systematic reviews of correlates of dietary behaviors specifically in 0- to 6-year-old children. We reviewed the quantitative evidence on determinants of early weaning and included 72 studies that examined 43 determinants; however, only six determinants were consistently associated with early weaning (young maternal age, low maternal education, low socio-economic status, absence or short duration of breastfeeding, maternal smoking, and lack of information and advice from healthcare providers) [23]. A review of the determinants of fruit and vegetable consumption among children and adolescents identified 98 papers and found that age, gender, socioeconomic position, preferences, parental intake and home availability/accessibility were important determinants/correlates of fruit and vegetable intake for all children under 18 years of age [29]. A review of determinants/correlates of children’s eating patterns and diet quality found that physical, social and family environments were important [30].

By including more recent studies, quantitative and qualitative studies, we aim to be able to draw more robust conclusions about the barriers and facilitators to be targeted in future interventions. Also younger children (0 to 3/4 years) may be influenced by different factors to those affecting 4- to 6-year-old children and, hence, it is important to study this entire age range. Indeed, there has been a recent increase in intervention studies targeting this younger age group [8,31-35] and it is important to include this age group in these reviews.

### Dissemination and plans for updating

The results will be disseminated to academic and non-academic audiences through peer-reviewed publications, conferences, formal presentations and in formal meetings.

Currently there are no plans for updating the reviews but this will be considered if a significant amount of new data becomes available.

## Abbreviations

ASSIA: Applied Social Sciences Index and Abstracts; BMI: Body mass index; BNI: British Nursing Index; EPPI: Evidence for Policy and Practice Information; HTA: Health Technology Assessment; PROSPERO: International Prospective Register for Systematic Reviews; RCT: Randomized control trial.

## Competing interests

The authors declare that they have no competing interests.

## Authors’ contributions

RL conceived the study and wrote the first draft. All authors contributed to designing the search strategy, commented on all aspects of the protocol, and approved the final version. HM carried out the database searches. VP, KH and CO carried out title and abstract screening. CS, RL and EvS contributed to quality assurance of the screening process. All authors read and approved the final manuscript.

## Supplementary Material

Additional file 1 Search Strategy. Click here for file

Additional file 2 Details of data extracted for different study designs. Click here for file
