# Supplementary material for: Protocol for systematic reviews of determinants/correlates of obesity-related dietary and physical activity behaviors in young children (preschool 0 to 6 years): evidence mapping and syntheses
Source: Syst Rev. 2013 May 10;2:28. doi: 10.1186/2046-4053-2-28 (PMC3691606; doi:10.1186/2046-4053-2-28)
Supplement: Additional file 1 — Search Strategy. [file 2046-4053-2-28-S1.pdf]

**Additional File 1: Search Strategy**

| | Medline and Embase via Ovid (01/08/2012) |
|---|---|
| 1 | (Determin*4 or correlates or factors or predict*3 or associate*3 or interaction or influence*1 or temperament or beliefs or attitudes or knowledge or perceptions or views or intentions or facilitators or barriers or experiences or prevent*3 or reduc*5 or increas*3 or promot*3 or education or curriculum or program*3 or polic*3 or media or campaign or review or intervention*1 or initiative*1 or strategy*3 or evaluation or trial).mp. [mp=title, abstract, original title, name of substance word, subject heading word, protocol supplementary concept, rare disease supplementary concept, unique identifier] |
| 2 | (Infant* or Toddler* or Preschool* or Nurser*).mp. [mp=title, abstract, original title, name of substance word, subject heading word, protocol supplementary concept, rare disease supplementary concept, unique identifier] |
| 3 | ((Fruit*1 or Vegetable*1 or juice or sugar sweetened beverage*1 or fizzy drinks or soft drinks or junk food or fast food or processed food or unhealthy food or takeaway food or non-core food or energy dense food or high fat food or fatty food or nutrient poor food or unhealthy diet or healthy eating or portion size or empty calories or confectionery or sweet*1 or dessert*1 or chocolate*1 or cake*1 or biscuit*1 or burger*1 or chip*1 or crisp*1 or snack*1 or breakfast or lunch or dinner or obes*6 or overweight).mp. [mp=title, abstract, original title, name of substance word, subject heading word, protocol supplementary concept, rare disease supplementary concept, unique identifier] |
| 4 | 3 not (allerg*3 or dental caries) |
| 5 | (physical activ*5 or inactiv*3 or exercise*1 or outdoor or TV or Television or Tele or sedentary or (screen adj time)).mp. [mp=title, abstract, original title, name of substance word, subject heading word, protocol supplementary concept, rare disease supplementary concept, unique identifier] |
| **6** | 1 AND 2 AND (4 OR 5) |
| **7** | 6 not (cerebral palsy or asthma or cystic fibrosis or autism).mp. [mp=title, abstract, original title, name of substance word, subject heading word, protocol supplementary concept, rare disease supplementary concept, unique identifier] |

| | Cinahl and Psychinfo via Ebsco (03/08/2012) |
|---|---|
| S1 | TX (Determin*4 or correlates or factors or predict*3 or associate*3 or interaction or influence*1 or temperament or beliefs or attitudes or knowledge or perceptions or views or intentions or facilitators or barriers or experiences or prevent*3 or reduc*5 or increas*3 or promot*3 or education or curriculum or program*3 or polic*3 or media or campaign or review or intervention*1 or initiative*1 or strategy*3 or evaluation or trial) |
| S2 | TX (Infant* or Toddler* or Preschool* or Nurser*) |
| S3 | TX (Fruit*1 or Vegetable*1 or juice or sugar sweetened beverage*1 or fizzy drinks or soft drinks or junk food or fast food or processed food or unhealthy food or takeaway food or non-core food or energy dense food or high fat food or fatty food or nutrient poor food or unhealthy diet or healthy eating or portion size or empty calories or confectionery or sweet*1 or dessert*1 or chocolate*1 or cake*1 or biscuit*1 or burger*1 or chip*1 or crisp*1 or snack*1 or breakfast or lunch or dinner or obes*6 or overweight) |
| S4 | TX S3 not (allerg*3 or dental caries) |
| S5 | TX (physical activ*5 or inactiv*3 or exercise*1 or outdoor or TV or Television or Tele or sedentary or (screen n1 time)) |
| S6 | TX S1 AND S2 AND (S4 OR S5) |
| S7 | TX S6 not (cerebral palsy or asthma or cystic fibrosis or autism) |

| | BNI via Healthcare Databases supplied by ProQuest (07/08/2012) |
|---|---|
| 1 | ((Determin*4 or correlates or factors or predict*3 or associate*3 or interaction or influence*1 or temperament or beliefs or attitudes or knowledge or perceptions or views or intentions or facilitators or barriers or experiences or prevent*3 or reduc*5 or increas*3 or promot*3 or education or curriculum or program*3 or polic*3 or media or campaign or review or intervention*1 or initiative*1 or strategy*3 or evaluation or trial)).ti,ab |
| 2 | ((Infant* or Toddler* or Preschool* or Nurser*)).ti.ab |
| 3 | ((Fruit*1 or Vegetable*1 or juice or sugar sweetened beverage*1 or fizzy drinks or soft drinks or junk food or fast food or processed food or unhealthy food or takeaway food or non-core food or energy dense food or high fat food or fatty food or nutrient poor food or unhealthy diet or healthy eating or portion size or empty calories or confectionery or sweet*1 or dessert*1 or chocolate*1 or cake*1 or biscuit*1 or burger*1 or chip*1 or crisp*1 or snack*1 or breakfast or lunch or dinner or obes*6 or overweight)).ti.ab |
| 4 | (allerg*3 or dental caries).ti.ab |
| 5 | 3 not 4 |
| 6 | (physical activ*5 or inactiv*3 or exercise*1 or outdoor or TV or Television or Tele or sedentary or (screen adj time)).ti.ab. |
| 7 | 1 AND 2 |
| 8 | 5 OR 6 |
| 9 | 7 AND 8 |
| 10 | (cerebral palsy or asthma or cystic fibrosis or autism).ti.ab. |
| 11 | 9 not 10 |

| | Assia and SocAbstracts via ProQuest (14/08/2012) |
|---|---|
| 1 | ab,ti(Determin*4 or correlates or factors or predict*3 or associate*3 or interaction or influence*1 or temperament or beliefs or attitudes or knowledge or perceptions or views or intentions or facilitators or barriers or experiences or prevent*3 or reduc*5 or increas*3 or promot*3 or education or curriculum or program*3 or polic*3 or media or campaign or review or intervention*1 or initiative*1 or strategy*3 or evaluation or trial) |
| 2 | ab,ti(Infant* or Toddler* or Preschool* or Nurser*) |
| 3 | ab,ti(Fruit*1 or Vegetable*1 or juice or sugar sweetened beverage*1 or fizzy drinks or soft drinks or junk food or fast food or processed food or unhealthy food or takeaway food or non-core food or energy dense food or high fat food or fatty food or nutrient poor food or unhealthy diet or healthy eating or portion size or empty calories or confectionery or sweet*1 or dessert*1 or chocolate*1 or cake*1 or biscuit*1 or burger*1 or chip*1 or crisp*1 or snack*1 or breakfast or lunch or dinner or obes*6 or overweight) |
| 4 | ab,ti(allerg*3 or dental caries) |
| 5 | S3 NOT S4 |
| 6 | ab,ti(physical activ*5 or inactiv*3 or exercise*1 or outdoor or TV or Television or Tele or sedentary or (screen near time)) |
| 7 | S1 AND S2 |
| 8 | S5 OR S6 |
| 9 | S7 AND S8 |
| 10 | ab,ti(cerebral palsy or asthma or cystic fibrosis or autism) |
| 11 | S9 NOT S10 |

| | Web of Knowledge via Thomson Reuters (13/08/2012) |
|---|---|
| 1 | ((Determin*4 or correlates or factors or predict*3 or associate*3 or interaction or influence*1 or temperament or beliefs or attitudes or knowledge or perceptions or views or intentions or facilitators or barriers or experiences or prevent*3 or reduc*5 or increas*3 or promot*3 or education or curriculum or program*3 or polic*3 or media or campaign or review or intervention*1 or initiative*1 or strategy*3 or evaluation or trial) AND (Infant* or Toddler* or Preschool* or Nurser*)) AND ((Fruit*1 or Vegetable*1 or juice or sugar sweetened beverage*1 or fizzy drinks or soft drinks or junk food or fast food or processed food or unhealthy food or takeaway food or non-core food or energy dense food or high fat food or fatty food or nutrient poor food or unhealthy diet or healthy eating or portion size or empty calories or confectionery or sweet*1 or dessert*1 or chocolate*1 or cake*1 or biscuit*1 or burger*1 or chip*1 or crisp*1 or snack*1 or breakfast or lunch or dinner or obes*6 or overweight) NOT (allerg*3 or dental caries) OR (physical activ*5 or inactiv*3 or exercise*1 or outdoor or TV or Television or Tele or sedentary or (screen near time))) |
| 2 | NOT (cerebral palsy or asthma or cystic fibrosis or autism) |
