# Supplementary material for: Protocol for systematic reviews of determinants/correlates of obesity-related dietary and physical activity behaviors in young children (preschool 0 to 6 years): evidence mapping and syntheses
Source: Syst Rev. 2013 May 10;2:28. doi: 10.1186/2046-4053-2-28 (PMC3691606; doi:10.1186/2046-4053-2-28)
Supplement: Additional file 2 — Details of data extracted for different study designs. [file 2046-4053-2-28-S2.pdf]

Additional File 2: Details of data extracted for different study designs

Observational/Non-intervention studies (cross-sectional and prospective)
Study id/pubmed id
Author
Year
Country
Age Range
Average age
Sex (Male: Female ratio)
Ethnicity
Socioeconomic status
Design (Cross sectional/Prospective)
Number of participants
Sample/recruitment e.g general population representative sample or specialist groups, Exposure/Determinant/Correlate examined
Measurement of Exposure/Determinant/Correlate (subjective or objective)
Measurement conducted by (researcher, parent etc)
Level of exposure/determinant/correlate- individual, family, childcare setting/preschool, community, policy/media/wider
Outcome (diet, physical activity, anthropometry - separate row for each outcome examined)
Measurement of outcome (self-report, objective)
Analysis (univariate or adjusted/multivariate)
Effect: ++ + 0 - --
Author email
Comments

Intervention Studies
Study id/pubmed id
Author
Year
Country
Age range
Design: cRCT, RCT, quasiRCT, before-after etc,
Number of participants,
Sample/recruitment e.g general population representative sample or specialist groups (deprivation, ethnicity, geography etc),
Intervention
Control
Level of intervention- individual, family, childcare setting/preschool, community, policy/media/wider
Theoretical model of intervention
Intervention provider
Number of sites
Outcome (diet, physical activity, anthropometry - separate row for each outcome examined), Measurement of outcome (self-report, objective)
Analysis
Effect- point estimate
Effect- Upper Confidence Interval
Effect- Lower Confidence Interval
Follow-up duration
Comments
Author email;
**Quality Assessment- score yes/no for each below (finally get total score)**
Randomisation
Effect of intervention reported for all outcomes

Pre intervention data on all outcomes
Post intervention data on all outcomes
Allocation concealment
Blinding
Objective measurement of outcome
Retention >70%

Qualitative studies
Study id/pubmed id
Author
Year
Country
Age
Design
Number of particpants
Sample/recruitment
Findings/direct quotes
Author conclusions
Comments
Author Email
**Quality Assessment- score yes/no (finally get total score)**
Research questions clearly stated
Approach appropriate for the research question
Qualitative approach clearly justified
Study context clearly described
Role of the researcher clearly described
Sampling method clearly described
Sampling strategy appropriate for the research question
Method of data collection clearly described
Data collection method appropriate
Method of analysis clearly described
Analysis appropriate for the research question
Conclusions supported by sufficient evidence
